# Falciparum malaria in young children of rural Burkina Faso: comparison of survey data in 1999 with 2009

**DOI:** 10.1186/1475-2875-10-296

**Published:** 2011-10-11

**Authors:** Claudia Beiersmann, Mamadou Bountogo, Justin Tiendrébeogo, Manuela De Allegri, Valérie R Louis, Boubacar Coulibaly, Maurice Yé, Olaf Mueller

**Affiliations:** 1 Institute of Public Health, Ruprecht-Karls-University Heidelberg, INF 324, 69124 Heidelberg, Germany; 2 Centre de Recherche en Santé de Nouna, BP 34, Nouna, Burkina Faso

## Abstract

**Background:**

Roll Back Malaria (RBM) interventions such as insecticide-treated mosquito nets (ITN) and artemisinin-based combination therapy (ACT) have become implemented with different velocities in the endemic countries of sub-Saharan Africa (SSA) in recent years. There is conflicting evidence on how much can be achieved under real life conditions with the current interventions in the highly endemic savannah areas of SSA.

**Methods:**

The study took place in a rural area of north-western Burkina Faso, which was defined as holoendemic in 1999. Clinical and parasitological data were compared in two cohorts of young children of the same age range from eight villages. Surveys took place in June and December of the year 1999 and 2009 respectively.

**Results:**

Prevalence of mosquito net use increased from 22% in 1999 to 73% in 2009, with the majority of nets being ITNs in 2009. In 2009, *P. falciparum *prevalence was significantly lower compared to 1999 (overall reduction of 22.8%).

**Conclusions:**

The reduction in malaria prevalence in young children observed between 1999 and 2009 in a rural and formerly malaria holoendemic area of Burkina Faso is likely attributable to the increase in ITN availability and utilization over time.

## Background

Malaria remains a major cause of global morbidity and mortality, with most of the burden being in sub-Saharan Africa (SSA) [1,2]. International attention to malaria has been revived in recent years, with the *Roll Back Malaria *(RBM) initiative coordinating global malaria control and elimination efforts [3]. Consequently and massively supported by various *Global Health Initiatives *(GHI), malaria control interventions - in particular insecticide-treated mosquito nets (ITN) and artemisinin-based combination therapy (ACT) - are currently rolled out in SSA on a large scale. However, the impact of these new developments on the malaria burden in the highly endemic areas of SSA continues to be hotly debated [2].

Here a systematic comparison of malaria survey data is presented, which were collected in 1999 and in 2009 in cohorts of young children of the same age range, from an endemic area of rural Burkina Faso.

## Methods

### Study area

Burkina Faso, a landlocked country in the heart of West Africa, is composed of 45 provinces divided into 13 administrative regions. The Kossi Province belongs to the Mouhoun Region and borders the Republic of Mali. Its administrative centre, Nouna town, is located 300 km north-west from Ouagadougou, the capital of the country.

The Kossi Province corresponds geographically to the Nouna Health District area, which has a size of 7464.44 km^2 ^and a population of about 320.000 in the year 2009 [4]. The area is a dry orchard savannah, inhabited mainly by subsistence farmers and cattle keepers of different ethnic groups. The short rainy season usually lasts from June to October [5]. The dry season includes two parts: a dry, cold and dusty period (November to February) and a dry and very hot period (March to May). The annual rainfall in this area varies between 500-1000 mm. Throughout the year, the mean minimum and maximum daily ambient temperatures are approximately 20°C and 40°C.

Formal health services in the district are restricted to the hospital in Nouna town and 29 village-based health facilities [4]. Malaria was defined as holoendemic in the area in 1999, with most transmission occurring during or shortly after the rainy season and 99% being attributed to *P. falciparum *[5]. The period July to December is considered as the high malaria transmission season, while the period January to June is considered as the low malaria transmission season. Access to malaria prevention has remained rather limited until very recently with only one quarter of households possessing ITNs in 2007 [6,7], but this proportion has increased to 59% in 2010 (De Allegri M., Louis V.R., Tiendrebéogo J., Souares A., Yé M., Tozan Y., Jahn A., Mueller O.: Universal Coverage with Malaria Control Interventions: Achievements and Challenges in Rural Burkina Faso, submitted). Access to quality malaria treatment was and is difficult in this rural study area [6,8], with only 15% of children with malaria having received early ACT in 2010 [9].

The fieldwork for this study was conducted in collaboration with the Nouna Health Research Center (CRSN), which is situated in the Kossi Province. The study area of the CRSN consists of Nouna town and 58 of the surrounding villages. A health and demographic surveillance system (HDSS) has been operating in the area since 1992 [4].

### Study design and procedures

This paper is part of a PhD project which compares systematically child health data from the year 1999 with corresponding data from the year 2009 in the Nouna area. The data from 1999 was collected within the frame of a large placebo-controlled community-based intervention trial, which looked at the efficacy of a zinc supplementation scheme on malaria morbidity and malnutrition [5,10,11]. In brief, 708 children aged 6-31 months from 18 villages were enrolled in June 1999 and closely followed up until December 1999. In addition, cross-sectional surveys took place in this study population in June and December 1999. Only children from the eight largest villages were included and only the data from the placebo arm of the zinc trial were used. The objective of this study was to compare malaria prevalence between 1999 and 2009.

In June 2009, children from the same eight villages and the same age range as in the zinc trial of 1999 were enrolled. They were randomly chosen from the HDSS register of the CRSN. Data collection in this children cohort took place through two cross-sectional surveys, conducted in June and December 2009. During surveys, a team consisting of two study physicians, study nurses, laboratory technicians and specifically trained field workers visited the villages. The study villages were Bourasso, Cisse, Kodougou, Koro, Nokui, Seriba, Sikoro and Solimana. The sample size calculation, which resulted in 460 children to be recruited in 2009, was based on expected differences between 1999 and 2009 in the primary outcomes (anthropometric variables) of the project. The chosen sample size allows for a detection of a reduction in malaria parasite prevalence of 15% with 80% power and at a significance level of 0.05.

For both study cohorts (1999 and 2009) data on various malaria parameters such as temperature, species-specific parasite rates and densities, malaria prevalence rates, as well as mosquito net utilization rates were collected. Data collection procedures for 1999 are described in detail elsewhere [5,11,12]. For 2009 a finger prick blood sample for thin and thick blood smears was collected by the laboratory technicians for malaria diagnosis. A questionnaire was applied by the trained fieldworkers to all mothers of study children, which - amongst other topics - addressed mosquito net possession and use as reported by the mother. During the surveys conducted in 1999 and 2009 malaria parasites were determined at the laboratory of the CRSN. Giemsa-stained thin and thick blood smears were examined by highly experienced technicians according to standard operation procedures established at the CRSN [5,13]. A 10% random sample of blood films is regularly cross checked at the laboratory of the Heidelberg School of Tropical Medicine.

### Data analysis

Data analysis was done using SAS version 9.2 (SAS Corporation, Cary, USA). Malaria was defined as ≥ 37.5°Celsius axillary temperature + ≥ 5000 *P. falciparum *trophozoites [5]. For comparisons (year 2009 to year 1999) a logistic regression model (SAS PROC LOGISTIC) was used for binary outcome variables (fever, malaria, *Plasmodium falciparum *prevalence) and a general linear model (SAS PROC GLM) was used for the continuous outcome variable (*Plasmodium falciparum *parasite density). The analyses were adjusted for age, sex, and village. Significance level was 0.05%.

### Ethical aspects

Ethical approval was obtained by the Ethical Committee of the Heidelberg University Medical School and the local authorities (Burkina Faso Ministry of Health in 1999 and local Ethical Committee in 2009). Prior to the study implementation, the trial was always explained in detail to and discussed with all relevant district authorities and the concerned communities. Community consent was sought. During the surveys, individual oral (in 1999) and written (in 2009) informed consent was sought from the respective caretakers of study children. The study children benefited from being treated free of charge in case of disease.

## Results

For the 1999 study, data were analysed for 179 and 197 children who took part in the June and December survey respectively. In 2009, data were analysed for 460 and 409 children who took part in the June and December survey respectively.

### Demographic data

Table 1 shows the demographic data from the 1999 and 2009 cohort children included into this study. Children recruited in June 2009 were slightly older compared to the children recruited in June 1999 (mean age 20.5 *vs*. 17.6 months, p ≤.0001). Moreover, there were small differences between 1999 and 2009 in the proportion of study children recruited from the eight study villages as well as in the reported ethnicity of participating children.

**Table 1:** Demographic characteristics of study children in 1999 and in 2009

		June 1999 N = 179		December 1999 N = 197		June 2009 N = 460		December 2009 N = 409	
		n	mean	n	mean	n	mean	n	Mean
**Age** **(months)**		**179**	**17.6**	**197**	**23.5**	**460**	**20.5**	**409**	**25.8**
		**N**	**%**	**N**	**%**	**N**	**%**	**N**	**%**

**Age** **Group**									
	**6-11 months**	31	17.3	-	-	64	13.9	-	-
	**12-23 months**	116	64.8	103	52.3	215	46.7	150	36.7
	**24-35 months**	32	17.9	91	46.2	181	39.4	231	56.5
	**36-47 months**	-	-	3	1.5	-	-	28	6.9
**Sex**									
	**Female**	94	52.5	107	54.3	218	47.4	184	45.0
	**Male**	85	47.5	90	45.7	242	52.6	225	55.0
**Village**									
	**Bourasso**	23	12.9	28	14.2	82	17.8	71	17.4
	**Cissé**	23	12.9	21	10.7	55	12.0	52	12.7
	**Kodougou**	20	11.2	22	11.2	24	5.2	18	4.4
	**Koro**	24	13.4	28	14.2	84	18.3	77	18.8
	**Nokui**	16	8.9	23	11.7	20	4.4	17	4.2
	**Seriba**	25	14.0	25	12.7	88	19.1	76	18.6
	**Sikoro**	22	12.3	25	12.7	54	11.7	48	11.7
	**Solimana**	26	14.5	25	12.7	53	11.5	50	12.2
**Ethnic** **Group**									
	**Bwaba**	41	22.9	52	26.4	129	28.0	113	27.6
	**Dafing**	71	39.7	72	36.6	202	43.9	179	43.8
	**Mossi**	28	15.6	34	17.3	63	13.7	54	13.2
	**Peulh**	26	14.5	28	14.2	57	12.4	50	12.2
	**Samo**	5	2.8	3	1.5	5	1.1	5	1.2
	**Other**	3	1.7	3	1.5	2	0.4	1	0.2
	**Missing**	5	2.8	5	2.5	2	0.4	7	1.7

### Malaria data

During 1999, reported mosquito net use of study children during the last night was rather low and averaged 20% (25% in the high malaria transmission season and 16% in the low malaria transmission season). In 2009, the corresponding figure was 73% (80% in the malaria transmission season and 66% in the non-malaria transmission season). While in 1999, none of the study children slept under an ITN, in 2009 the great majority of study children's mosquito nets (72%) were ITNs.

Additional file 1, Table S1 shows the comparison of clinical and parasitological data between the 1999 and 2009 study children, after adjustment for age, sex, and village. Fever and malaria prevalence was consistently lower during the June compared to the December surveys. There were no significant differences in fever (≥37.5°C axillary's temperature) prevalence and falciparum malaria (≥37.5°C axillary's temperature + ≥5.000 asexual *P. falciparum *parasites/μl) prevalence between the corresponding survey months in 1999 and 2009, but *P. falciparum *prevalence was significantly lower in the 2009 compared to the 1999 surveys. There was an overall 22.8% reduction in *P. falciparum *prevalence between 1999 and 2009.

## Discussion

The main finding from this study is a significant reduction in *P. falciparum *parasite prevalence in 2009 compared to 1999, although overall parasite prevalence remained above 50% in this population of young children in rural SSA. This development is most likely attributable to an increase in mosquito net and in particular ITN utilization by young children in the area. Major reductions in the rates of malaria disease and all-cause childhood mortality have consistently been associated with the use of ITNs in the endemic areas of SSA, and this has recently been shown to be of comparable magnitude under programme conditions [2,14]. However, only few data are available about the effects of ITN use on malaria parasite prevalence. The overall reduction in parasite prevalence associated with the ITN intervention in randomised controlled trials has been shown to be only 13% in a Cochrane review [15]. Recently published data from six malaria indicator surveys in SSA arrive at a pooled reduction in parasite prevalence of 24% associated with the ITN intervention [14]. In these six countries, ITN household coverage ranged from 27% to 58%, which is close to the 59% ITN household coverage known from the most recent survey in the Nouna study area. Thus, the findings from this study of an overall parasite prevalence reduction of 23% support similar findings from a number of other countries in SSA.

However, in 2009 ITN coverage was still below the RBM target of 80% in this population of rural Burkina Faso. It remains to be seen how malaria parameters will develop after universal coverage has been achieved [3,16]. At the end of 2010 the national malaria programme of Burkina Faso started a mass distribution of ITNs to the whole population of the country with the aim to have one ITN per two household members [17]. In addition, access to ACT through governmental health facilities has been in place since 2007 and is now planned to be supported by village health worker-based syndromic ACT treatment of fever cases. Thus, further surveys will demonstrate what can finally be achieved with the current rollout of RBM interventions in a formerly holoendemic area of West African savannah.

This study has several strengths and limitations. First, it systematically compares malaria parameters in a large sample of young children over a period of ten years, before and after the roll-out of ITN programme in the country. However, it only compares data from two years and the findings may thus be influenced by unknown confounding variables such as weather conditions. Second, the two study cohorts were carefully matched for key socio-demographic parameters such as age, sex and village. However, they still differed slightly in a number of parameters, and even after adjustment in the analysis for known confounders, there might have remained some residual confounding. Third, there might also have been slight differences in the performance of examinations during the 1999 and 2009 surveys, as different study teams were in place. Although an influence of this cannot be ruled out, the parasitological results are likely not to have been affected much as there are standard operation procedures for malaria diagnosis in the laboratory of the CRSN. It also has to be considered that the study only shows malaria prevalence developments in eight purposively selected villages which can't be generalised to the whole Nouna Health District or to the whole country. Finally, there were many developments in the study area over the last 10 years, such as improvements in access to health services and in nutrition indicators, which may all have some influence on the local malaria burden.

## Conclusions

The findings from this study show a significant reduction in malaria parasite prevalence in young children between 1999 and 2009 in a rural and formerly malaria holoendemic area of Burkina Faso, which is likely attributable to the increase in ITN utilization over time.

## Competing interests

The authors declare that they have no competing interests.

## Authors' contributions

CB contributed to the conception and design of the study, developed the questionnaire, collected the data, analysed the data and wrote the paper. MB contributed to the conception and design of the study, developed the questionnaire, collected the data, and contributed to writing the paper. JT contributed to developing the questionnaire, collected the data and contributed to writing the paper. MA contributed to the conception and design of the study, developing the questionnaire, contributed to analysis and interpretation of the data and to writing the paper. VL contributed to designing the study and to writing the paper. BC contributed to designing the study and to writing the paper. MY contributed to designing the study and to writing the paper. OM initiated the conception and design of the study and contributed to interpretation of the data and to writing the paper. All authors read and approved the final manuscript.

## Supplementary Material

Additional file 1 **Table S1: Comparison of clinical and parasitological survey data in 1999 and in 2009**. Additional file 1 contains a table (Table S1) that shows the comparison of clinical and parasitological survey data between the 1999 and 2009 study children, after adjustment for age, sex, and village. Click here for file
