# Supplementary material for: Falciparum malaria in young children of rural Burkina Faso: comparison of survey data in 1999 with 2009
Source: Malar J. 2011 Oct 11;10:296. doi: 10.1186/1475-2875-10-296 (PMC3200185; doi:10.1186/1475-2875-10-296)
Supplement: Additional file 1 — Table S1: Comparison of clinical and parasitological survey data in 1999 and in 2009. Additional file 1 contains a table (Table S1) that shows the comparison of clinical and parasitological survey data between the 1999 and 2009 study children, after adjustment for age, sex, and village. [file 1475-2875-10-296-S1.PDF]

Table S1: Comparison of clinical and parasitological survey data in 1999 and in 2009

	June 1999 (N=179)		June 2009 (N=460)		Comparison*		December 1999 (N=197)		December 2009 (N = 409)		Comparison*	
	n	%	n	%	OR (95% CI)	p-value	n	%	n	%	OR (95% CI)	p-value
**Fever**[1]	7	3.9	29	6.3	1.94 (0.78 – 4.80)	0.1522	22	11.2	33	8.1	0.85 (0.46 – 1.57)	0.5960
**Malaria**[2]	2	1.1	6	1.3	-	-	13	6.6	13	3.2	0.65 (0.27 – 1.58)	0.3460
**Plasmodium falciparum prevalence**												
**≥ 1**	113	63.1	174	37.8	0.26 (0.17 – 0.39)	<.0001	169	85.8	268	65.5	0.26 (0.16 – 0.43)	<.0001
**≥ 5000**	16	8.9	21	4.6	0.37 (0.18 – 0.76)	0.0067	78	39.6	89	21.8	0.44 (0.30 – 0.65)	<.0001
**≥ 100000**	0	0	0	0	-	-	3	1.5	-	-	-	-
**Missing**	5	2.8	7	1.5	-	-	4	2.0	4	1.0	-	-
	**N**	**Mean (95% CI)**	**N**	**Mean (95% CI)**	**p-value (95% CI)**		**N**	**Mean (95% CI)**	**N**	**Mean (95% CI)**	**p-value (95% CI)**	
**Plasmodium falciparum parasite density/µl**	113	2482 (1103; 3860)	174	2994 (1773; 4216)	0.5811 (-2340; 1315)		169	9460 (7413; 11507)	268	6521 (4780; 8263)	0.0324 (247; 8263)	

[1] defined as ≥ 37.5° Celsius
[2] defined as ≥ 37.5° Celsius AND ≥ 5000 trophozoites
*adjusted for age, sex and village
